# Associations between maternal occupational exposures and pregnancy outcomes among Chinese nurses: a nationwide study

**DOI:** 10.1186/s12978-023-01704-x

**Published:** 2023-10-31

**Authors:** Zhaoqiang Jiang, Junfei Chen, Lingfang Feng, Mingying Jin, Shuang Liu, Lina Wang, Jing Wang, Changyan Yu, Jianhong Zhou, Yan Ye, Liangying Mei, Wenlan Yu, Xing Zhang, Jianlin Lou

**Affiliations:** 1 School of Public Health, Hangzhou Medical College, 182 Tianmushan Road, Hangzhou, 310013 China; 2 School of Medicine, The First Affiliated Hospital, Huzhou University, Huzhou, Zhejiang Province China; 3 Chinese Center for Disease Control and Prevention, National Institute of Occupational Health and Poison Control, Beijing, China; 4 Department of Gynecology, Women’s Hospital, Zhejiang University School of Medicine, Xueshi Road, Hangzhou, 310006 China; 5 Department of Occupational Health, Beijing Center for Diseases Prevention and Control, Beijing, China; 6 Hubei Provincial Center for Disease Control and Prevention, Wuhan, China

**Keywords:** Female, Nurses, Occupational exposure, Pregnancy outcome

## Abstract

**Background:**

Several studies have provided evidence about adverse pregnancy outcomes of nurses involved in occupational exposure. However, the pregnancy outcomes among nurses in middle-income countries are not well demonstrated. The main aim of this study is to present the prevalence and influencing factors of pregnancy outcomes among female nurses in China.

**Methods:**

We included 2243 non-nurse health care workers, and 4230 nurses in this national cross-sectional study in China. Information on occupational exposures and pregnancy outcomes was collected using a face-to-face investigation. Odds ratios (*ORs*) were estimated through logistic regression.

**Results:**

The proportion of threatened abortion, spontaneous abortion, and stillbirth of female nurses was 2.6%, 7%, and 2.1%, respectively. We found an increased risk of threatened abortion among nurses with overtime work (*OR* = 1.719*, *95% CI 1.158–2.550). The risk of threatened abortion and spontaneous abortion was elevated among nurses handling disinfectant (*OR* = 2.293 and 1.63, respectively). We found a nearly twofold increased risk of premature birth (*OR* = 2.169, 95% CI 1.36–3.459) among nurses handling anti-cancer drugs.

**Conclusions:**

Our findings suggested that maternal occupational exposures might be associated with the risk of adverse pregnancy outcomes among female nurses in China. We recommend that policy-markers and hospital managers work together to reduce exposure to occupational hazards and improve pregnancy outcomes among female nurses.

**Supplementary Information:**

The online version contains supplementary material available at 10.1186/s12978-023-01704-x.

## Introduction

Adverse pregnancy outcome is a wide range of diseases, including but not limited to threatened abortion, spontaneous abortion, premature birth, stillbirth, and low birth weight [1]. The nursing profession is one of the largest work-forces in health care professionals, with more than 75% of female nurses of reproductive age [2]. Therefore, one of the most remarkable fields of investigation is the pregnancy outcome of female nurses. Valanis et al. [3] found a combined risk of spontaneous abortion and stillbirth among 2976 female nurses and pharmacists. A recent study [2] also found a higher risk of preterm delivery during the perinatal period among nurses.

Occupational hazards, including exposure to anesthetic gas [4], anti-cancer drugs [5], disinfectants [6], night shift work [7], overtime work [8], and prolonged standing [9], were common among nurses worldwide. Occupational hazards are the main risk factors that may affect pregnancy outcomes of female nurses [10]. According to a previous study, which involved 8461 female nurses in the USA, antineoplastic drug exposure was related to a twofold increased risk of spontaneous abortion, while sterilizing agents exposure with a twofold increased risk of late spontaneous abortion (12–20 weeks) [11]. Matte [12] found that the offspring of nurses who were exposed to occupational hazards during pregnancy had an increased risk of prenatal development of congenital anomalies. Moreover, a high risk of congenital anomalies was found to be related to exposure to anesthetic gases, including halogenated gases and nitrous oxide [13].

A study in the Netherlands demonstrated that spontaneous abortion, stillbirth, and congenital anomalies were not related to antineoplastic drug exposure [14]. A recent meta-analysis, including 24 published epidemiological studies of nurses, found an increased risk of adverse pregnancy outcomes [15]. However, the strength of association was rated as weak (odds ratio < 1.5). Arbour [16] found that the offspring of registered nurses in Canada had a lower prevalence of congenital anomalies and low birth weight than the general population.

There are more than 4450 thousand registered nurses in China [17]; however, previous studies in China rarely investigated and reported pregnancy outcomes of female nurses. A study in southern China, involving a small sample of 473 female nurses, found that shift work could increase the prevalence of menstrual cycle irregularity [18]. However, this study only focused on menstrual disorders.

There is a gap between numerous occupational hazards and inadequate health protection policies in healthcare institutions [19], especially in small and medium-sized hospitals in China. Hospital managers urgently need new findings to develop and implement protection strategies to manage occupational hazards and improve working conditions among nurses. Furthermore, institutional support and protection of nurses could improve the care quality of female nurses, and women’s empowerment collectives could increase the responsiveness of healthcare providers [20]. Hence, the present study aims to investigate the prevalence of adverse pregnancy outcomes among female nurses in China and explore the association between work-related hazards and adverse pregnancy outcomes. Finally, this cross-sectional study will provide novel findings and potential strategies for healthcare providers to address the gap between occupational hazards and health protection policies in healthcare institutions.

## Materials and methods

### Study design

This nationwide study was conducted in 2016 in China, using a cross-sectional study design. To guarantee the representation of the study population, we included female medical staff from fifteen provinces in China, covering north, south, east, west, and central China. We applied a convenience sampling method in each province to select medical institutions. Then, female medical staff in these medical institutions were randomly selected. We selected female nurses in these medical institutions as the studying group and chose other female non-nurse medical staff in the same medical institutions as the control group. The control group included physicians, surgeons, pharmacists, hospital administrators, rehabilitation therapists, and other workers in medical institutions.

To ensure the investigation quality, we gathered experts in occupational health, reproductive health, humanities research, and legal research to form an expert panel. The panel of experts demonstrated and modified the project plan, provided guidance for the entire on-site investigation, and participated in project investigations. Project members had a regular meeting every three months to discuss project progress.

### Sample size calculation

First of all, we calculated the sample size using the formula based on the simple sampling method. We focused on female nurses’ pregnancy outcomes, such as low-weight birth, stillbirth, premature birth, threatened abortion, and spontaneous abortion. We used a proportion of adverse pregnancy outcomes (34%) for sample size calculation [21] and set the allowable error rate as 5%. Then we got an estimated sample of 344 in each province and 5172 in fifteen provinces. Considering sampling error, we enlarged the sample size by dividing the calculated sample size by 0.8 and got an estimated sample size of 6465.

### Inclusion and exclusion criteria

The inclusion criteria were: (1) female medical staff working in health care settings; (2) aged 18–50 years old; (3) able to communicate; (4) agree to provide information through a questionnaire and accept to participate in the study; and (5) women who self-reported as sexually active. The exclusion criteria were: (1) individuals whose ages were beyond 18–50 years; (2) those with missing data on occupational exposure and menstrual characteristics; and (3) those without pregnancy information.

### Study population

**Fig. 1:**
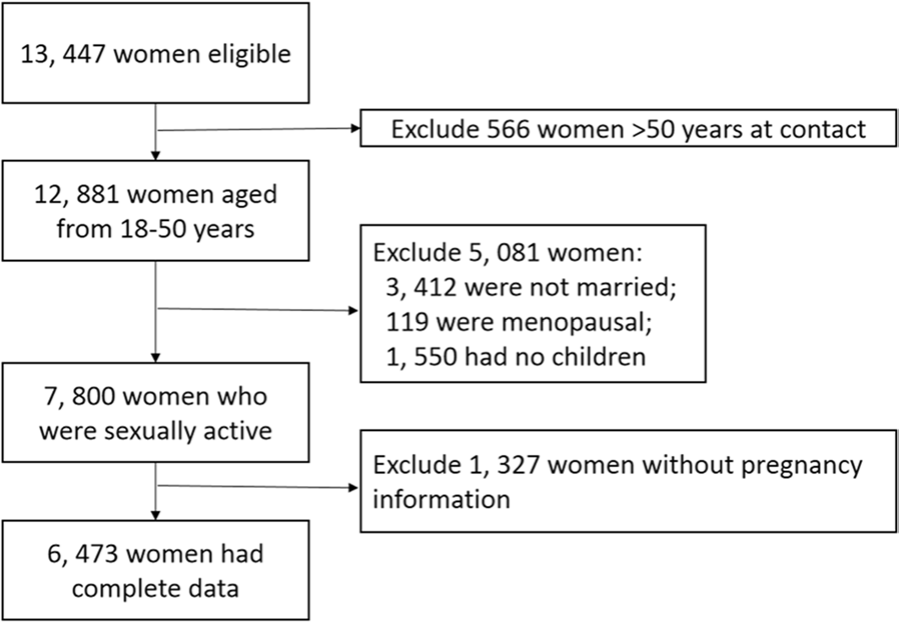
Flow chat

According to the inclusion and exclusion criteria, 2243 non-nurse healthcare workers and 4, 230 nurses from 1300 medical health institutions were included in this study (Fig. 1). Among 6473 participants, 5493 (84.9%) were from hospitals, 897 from community health care centers, 27 from scientific research institutions, and 56 from commercial pharmacy institutions. The response rate was 83% (6473/7800) among those who were sexually active. More than 820 investigators participated in the study, and 10 of them were cadre investigators.

### Exposure assessment

Well-trained investigators conducted a structured face-to-face investigation using a self-designed questionnaire in each medical health institution from March 1st to December 31st, 2016. This questionnaire included demographic information, occupational activities, and self-reported pregnancy outcomes. Occupational histories, including occupational work styles and occupational exposures, were self-reported. For example, the investigator asked “Have you ever worked overtime?” and explained to the participant that working overtime meant working more than 8 h per day. The participant answered “yes” or “no”. Then the investigator ticked and coded the option on the questionnaire. Besides, the definition of occupational histories was reported in a previous article [22]. In brief, we defined vibration exposure (also known as “whole body vibration”) as “the mechanical vibration that, when transmitted to the whole body, entails risks to the health and safety of workers, in particular, lower-back morbidity and trauma of the spine” [23].

### Outcomes of interest

**Fig. 2:**
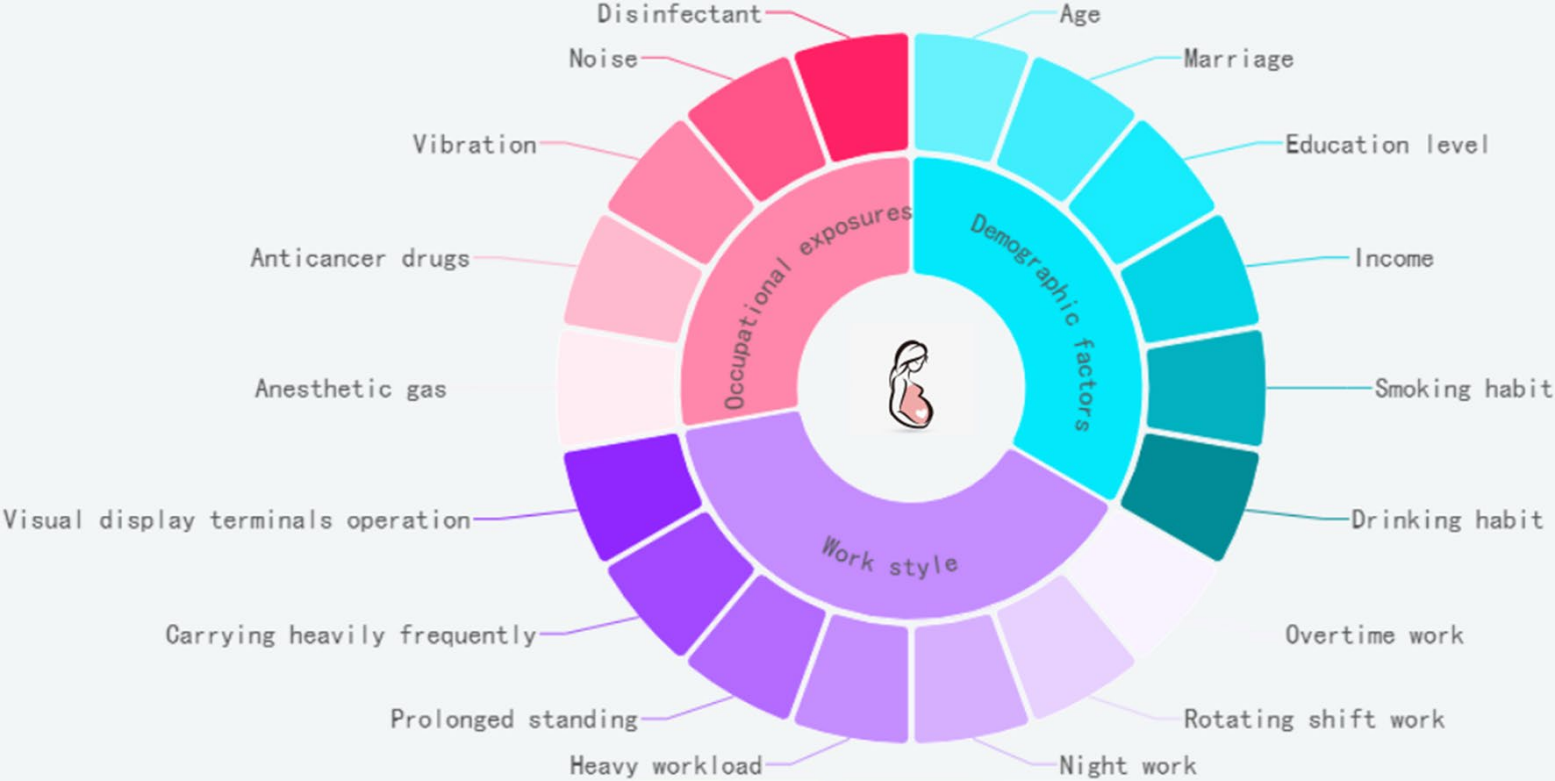
Theoretical framework showing the possible factors which influence the occurrence of adverse pregnancy outcomes

We defined infertility as the failure to achieve a clinical pregnancy after 12 months or more of regular unprotected sexual intercourse [24]. Spontaneous abortion was defined as a pregnancy loss before 20 weeks [25]. Low birth weight was defined as offspring weighing less than 2, 500 g [26], and premature birth was defined as offspring born at a gestational age of fewer than 37 weeks [27]. To clarify the theoretical framework, we drew a figure to illustrate the possible factors influencing adverse pregnancy outcomes (Fig. 2).

### Ethical considerations

The current study was conducted under the Declaration of Helsinki. We obtained written informed consent from all the participants. The study was approved by the Zhejiang Academy of Medical Sciences (Ethical Review Research No. 10, 2013).

### Statistical analysis

Normal distribution was examined using the Kolmogorov–Smirnov test. Frequency and proportion were used to determine the prevalence of adverse pregnancy outcomes. For categorical variables such as occupational factors and adverse pregnancy outcomes, the Chi-squared test was used for determining the difference between the proportions. Pearson's chi-square test is a statistical method to test if there is a difference between samples of data. It is a procedure for testing hypotheses when the data are categorical [28]. Logistic regression analysis was used to assess the relationship associations between influencing factors and the risk of adverse pregnancy outcomes, based on the backward method. Age, marriage, income, and education level were set as control variables in the logistic regression models. Because very few people had smoking or drinking habits, we did not include them in the model. The results of regressions were shown by odds ratios (*OR*s) with 95% confidence intervals (*CI*s). Two-sided p < 0.05 was considered to be statistically significant. Statistical analyses were performed using R software (version 3.4.4).

## Results

### Sociodemographic characteristics

Female nurses were younger than female non-nurse healthcare workers (Table 1). The total income of nurses was significantly lower than non-nurse healthcare workers. Smoking habit was more popular among non-nurse healthcare workers than that among nurses, with percentages of 1.1% and 0.4%, respectively. Nurses with an associate degree or above accounted for 94.7% of the total nurse population.

**Table 1:** Sociodemographic characteristics of nurses and non-nurse health care workers

Variables	Categories	Non-nurse workers *n* = 2243	Nurses *n* = 4230	*Z*/χ^2^	*P*
Age, years [median (IQ)]	–	37 (10)	34 (10)	188.75^a^	< 0.01
Marriage, *n* (%)	Married	2180 (97.2)	4118 (97.4)	0.14^b^	0.70
	Divorce or widowed	63 (2.8)	112 (2.7)		
Education level, *n* (%)	Below associate degree	116 (5.2)	225 (5.3)	0.06^b^	0.80
	Associate degree or above	2127 (94.8)	4005 (94.7)		
Income, *n* (%)	< 10, 000 yuan	189 (8.4)	429 (10.1)	17.56^b^	< 0.01
	10, 000–50, 000 yuan	833 (37.1)	1645 (38.9)		
	50, 000–100, 000 yuan	912 (40.7)	1510 (35.7)		
	> 100, 000 yuan	309 (13.8)	646 (15.3)		
Smoking habit, *n* (%)	–	25 (1.1)	16 (0.4)	12.63^b^	< 0.01
Drinking habit, *n* (%)	–	58 (2.6)	99 (2.3)	0.37^b^	0.54

IQ: interquartile rage

^a^Two-sample Wilcoxon test was used

^b^chi-square test was used

### Nurses are exposed to more occupational hazards

**Fig. 3:**
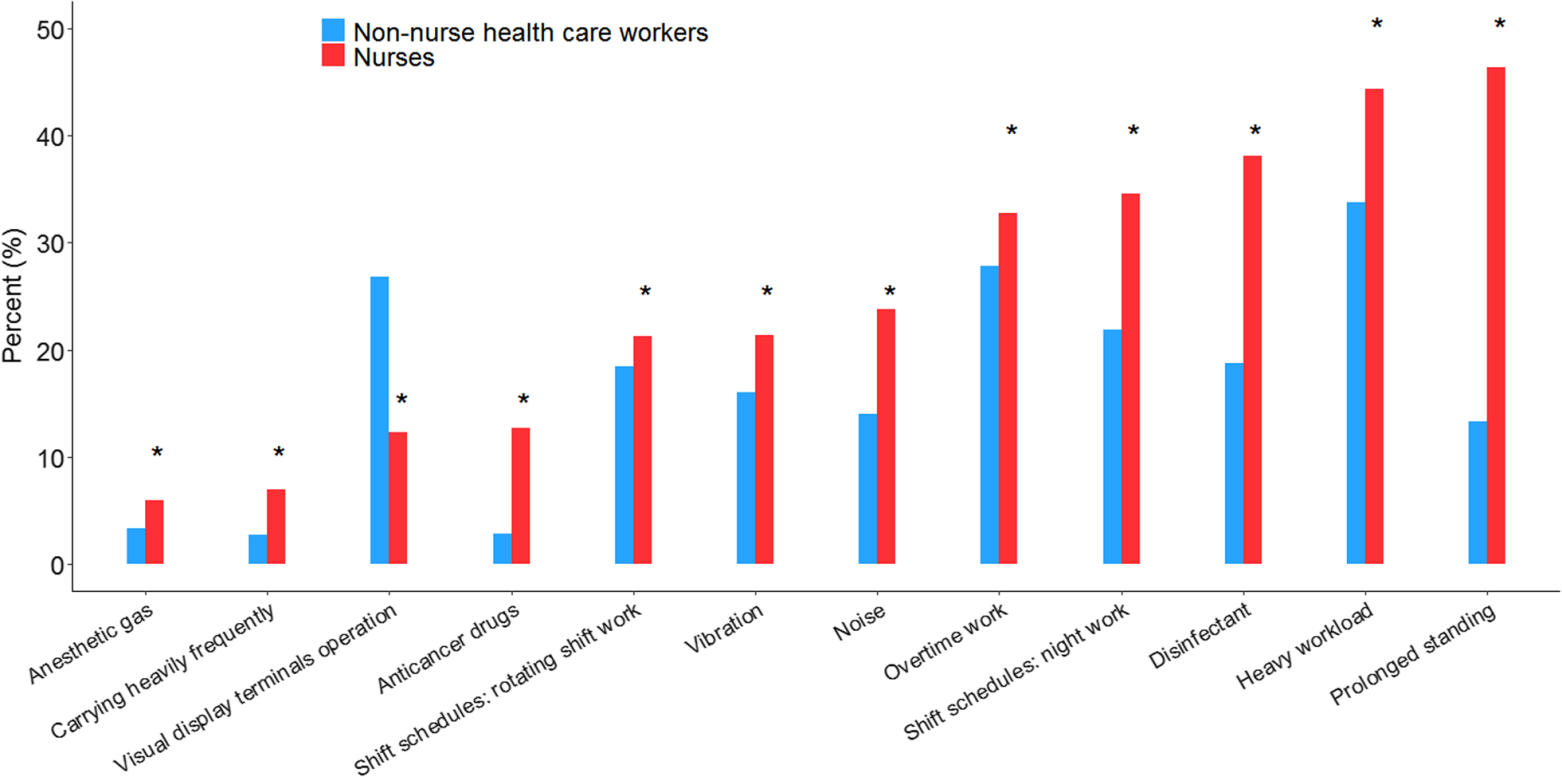
Occupational hazards between nurses and non-nurse health care workers. *p < 0.05, compared with non-nurse healthcare workers

In almost every occupational hazard except for the noise and visual display terminal operation, the exposure proportions to occupational hazards were higher among nurses than among non-nurse health care workers (Fig. 3). The occupational hazard of prolonged standing or carrying heavily frequently was the leading occupational hazard of nurses. Of 4230 nurses, 1946 nurses (46.3%) stood for a long time, and 7% carried heavily frequently. Adverse work styles such as heavy workload, night work, and overtime work were common among nurses, with proportions of more than 30%. The rate (38%) of nurses exposed to disinfectants was higher than that (18.7%) of non-nurse healthcare workers. The rate of nurses was 34.5% for those who had night work, while the rate was 21.3% for shift work. The proportions of nurses exposed to anti-cancer drugs or anesthetic drugs were 12.7% and 6%, respectively, higher than non-nurse healthcare workers. Controversially, non-nurse healthcare workers were more likely to be exposed to noise and operate visual display terminals than nurses.

### Nurses are more likely to have abortions or stillbirths

**Fig. 4:**
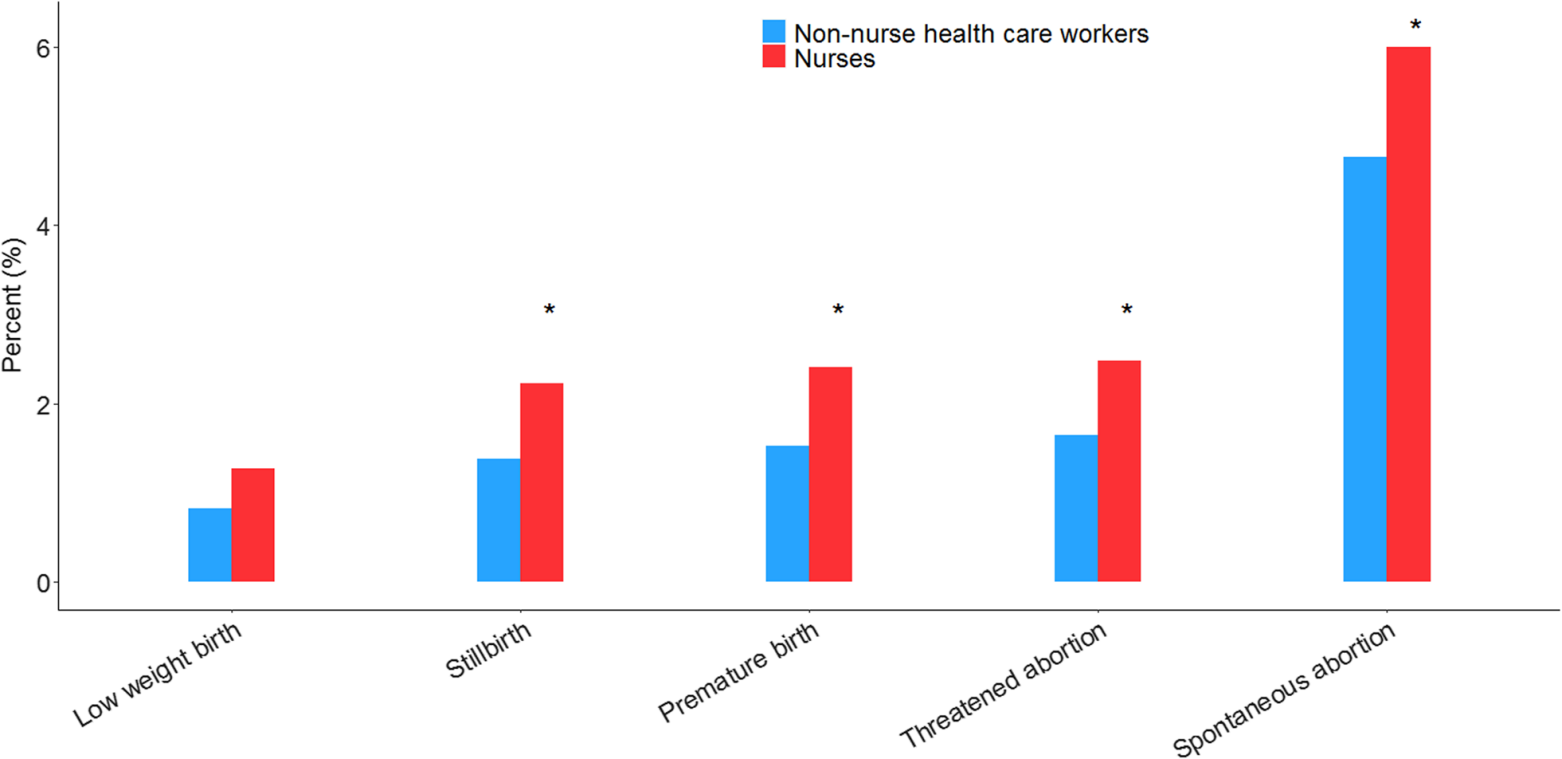
Adverse pregnancy outcomes between female nurses and non-nurse healthcare workers [*n* (%)]. *p < 0.05, compared with non-nurse healthcare workers

The statistical analysis showed a significant difference in the prevalence of threatened abortion, spontaneous abortion, and stillbirth. For nurses, the proportion of threatened abortion and spontaneous abortion was 2.5% and 6%, respectively, which were higher than those of non-nurse healthcare workers (1.7% and 4.8%, respectively; Fig. 4). Of all the nurses, 2.4% experienced stillbirth, which was significantly higher than that of non-nurse healthcare workers (1.5%). We found no significant differences in the prevalence of premature birth and low-weight birth in the two groups.

### Occupational exposures to nurses are associated with adverse pregnancy outcomes

**Fig. 5:**
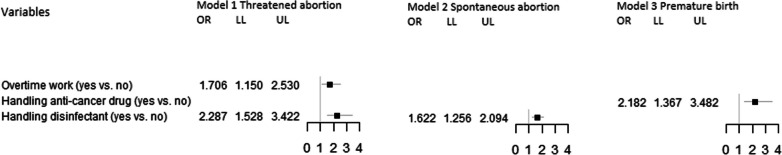
Association between occupational hazards and adverse pregnancy outcomes among female nurses. Adjusted regression models were displayed, controlling for age, marriage, and education level; LL: lower level of 95% confidence interval; UL: upper level of 95% confidence interval

We focused on the adverse pregnancy outcome of nurses and found an increased risk of threatened abortion among nurses with overtime work (*OR* = 1.706, 95% CI 1.15–2.53; Fig. 5). Threatened abortion was also positively associated with handling disinfectants among nurses (*OR* = 2.287, 95% CI 1.528–3.422). Spontaneous abortion was associated with handling disinfectants among nurses (*OR* = 1.622, 95% CI 1.256–2.094). We found a nearly twofold elevated risk of premature birth (*OR* = 2.182, 95% CI 1.367–3.482) among nurses handling the anti-cancer drugs.

### Sensitivity analysis

To assess the robustness of the findings, we performed sensitivity analyses by excluding 980 nurses from primary medical and health institutions to minimize potential bias due to the medical institution level. The results did not materially change (Additional file 1: Fig. S1). However, among hospital nurses, prolonged standing or frequent heavy lifting was positively associated with threatened abortion (*OR* = 1.497, 95% CI 1.035–2.165). Nurses under noise exposure had a 1.497-fold elevated risk for threatened abortion and a 1.355-fold elevated risk of spontaneous abortion.

## Discussion

As far as we know, our findings reported the adverse pregnancy outcomes of female nurses in China for the first time. The studying population was a representative sample of nurses in China. Our national survey found that sexually active nurses had more occupational hazards than non-nurse healthcare workers. The results of our study provided evidence that occupational hazards to nurses could lead to increased adverse pregnancy outcomes such as threatened abortion, spontaneous abortion, and stillbirth.

Nurses were exposed to many occupational hazards. The present study found that nurses were exposed to anesthetic gas, anti-cancer drugs, and disinfectants more frequently than non-nurse healthcare workers. The discrepancy in exposure rate could be explained by the fact that nurses were licensed to provide clinical services, such as injections, ward rounds, dispensing, and disinfection. These jobs exposed nurses to chemical reagents, biological pathogens, and physical damage. In particular, we observed a significantly higher frequency of anti-cancer drug exposure (13.7%) among nurses than among non-nurse healthcare workers, which was in line with the facts of nurses’ jobs. Anti-cancer drugs, also called antineoplastic drugs, are known to be carcinogenic to human beings [29]. Anti-cancer drugs could be found on the workplace surface and in the air in drug preparation rooms, which may cause dermal exposure among nurses who handle the drugs. A multi-center study in Canada [30] found that 43.4% of the sampled sites in 66 hospitals were contaminated with cyclophosphasmide, which is somewhat similar to our findings. Our findings suggestthat nurses in China are occupationally exposed to a certain amount of anti-cancer drugs, which might cause adverse health effects those handling anti-cancer drugs.

Here, we wanted to compare the proportion of each adverse pregnancy outcome of nurses with the previous study and reveal how the distribution of adverse pregnancy outcomes was among nurses in China. First, our study found that 2.6% of nurses had experienced threatened abortion, much lower than the rate (20%) in the previous report [31]. Generally speaking, nearly half of pregnant nurses suffered from threatened abortion [32]. However, the symptoms and outcomes of threatened abortion are usually not severe, and nurses may forget them over a long time. It may lead to an underestimation of the threatened abortion prevalence. Meanwhile, the discrepancy in threatened abortion proportion between different studies might be partly due to the potential recall bias by questionnaire surveillance, related to the length of recall [33]. Secondly, the prevalence of spontaneous abortion in our study was higher than that reported in a national study on 3.9 million women in China [34], and lower than that in the Nurses' Health Study II (18.8%) [35]. Thirdly, the preterm birth rate in our study was 2% among female nurses, lower than that reported in the general population of the United Kingdom (5.83%). Fourthly, the stillbirth proportion among nurses was 2.1%, a little higher than that in Brazil (1.5%) [36]. Generally, the prevalence of adverse pregnancy outcomes varied between different studies, and the investigation information might be biased. However, the pregnancy outcomes regarding birth or death were always accurate by the investigation information from mothers, as the birth or death of an embryo was a significant event for the mother. Hence, we could speculate from these results that adverse pregnancy outcomes might be related to the harmful occupation feature of the nurse.

We found that overtime work was associated with threatened abortion, observed in the previous study [37]. As is known to all, most nurses worked overtime and in shift work. Nurses were predominantly scheduled for 12-h shifts, and nearly half work weekly overtime, and that trend has remained relatively stable over the past ten years [38]. A study in China [39] found that 65.5% of medical oncology staff members worked for long working hours, while 77% were female. In this situation, overtime work was associated with fatigue and psychological stress [40]. The underlying mechanism might be that stress could result in the continuous activity of the sympathetic nervous system and hypothalamic–pituitary–adrenal (HPA) axis [3]. Then stress-related biomarkers, such as corticosterone, serotonin transporters, dopamine-beta-hydroxylase, and brain-derived neurotrophic factor (BDNF), are released and might affect the ovary [41]. Another possible explanation is that acute stress could inhibit progesterone release [42], while inadequate progesterone in early pregnancy could lead to miscarriage [43]. Therefore, hospital administrators should recognize the potential effects of overtime work on reproductive health and endeavor to develop new methods to improve the working situation.

The elevated association between handling disinfectants and the risk of threatened abortion and spontaneous abortion was consistent with previous toxicological research in the United States [44]. Animal experiments found that disinfectants such as ethylene oxide could increase feral loss in pregnant animals [45]. Furthermore, a previous study in South Africa [46] found a significantly increased risk of spontaneous abortion (*OR* = 20.8, 95% CI 2.1–199) with ethylene oxide. Hence, our data implicated disinfectants as a possible reproductive toxicant among female nurses in China. Disinfection byproducts exposure through the skin or respiratory tract might account for the majority of occurrences of spontaneous abortion. However, we should apply a detailed analysis of the association between each type of disinfectant and abortion in future studies.

We found a nearly twofold increased risk of premature birth among nurses who handled anti-cancer drugs, similar to a previous study [14]. Anti-cancer drugs, also called cytotoxic (antineoplastic) drugs, had reproductive toxicity. The anti-cancer drugs could interact with nucleic acids from the cell, inhibit DNA synthesis, and cause genotoxic damage in nurses [47]. Nurses were exposed to anti-cancer drugs when preparing or administrating the drugs. However, the detailed mechanism of the anti-cancer drug for premature birth is unknown. Therefore, nurses should fill prescriptions in the ventilation hood to avoid drug exposure via the skin, and avoid contact with anti-cancer drugs during pregnancy. Our findings might benefit hospital managers to apply information and communications technologies to manage anti-cancer drugs more efficiently.

Although the sensitivity analysis showed similar results as the primary analysis, our research produced some interesting findings. We found that prolonged standing or frequent heavy lifting increased the risk of threatened abortion among hospital nurses. It could be explained by the heavy workload of nurses in the hospitals. A recent systematic review and meta-analysis [48] supported our findings and indicated that working with heavy lifting, prolonged standing, or heavy physical workload could increase the risk of spontaneous abortion. Hence, we recommend hospital managers lowering nurses' physical workload during their pregnancies and reducing the risk of ergonomic factors on threatened abortion.

Our results from sensitivity analysis also indicated that noise exposure was associated with threatened abortion and spontaneous abortion among nurses in the hospital. Noise exposure in the hospital mainly comes from various instruments and equipments. In addition, there are different type of noises in hospitals (e.g., babies crying, talking loudly, or shouting) [49]. Animal studies have shown that noise might increase embryo absorption and decrease live births [50]. The underlining mechanism might be related to responses of the nervous systems and the brain evoked by noise exposure. These responses elicit changes in endocrine function and decreased uteroplacental blood flow [51], leading to abortion. However, further research is needed to clarify the mechanism between noise exposure and the risk of threatened abortion and spontaneous abortion.

The present study had many notable strengths. Up to our current knowledge, our study examined several kinds of occupational exposures with the subsequent risk of adverse pregnancy outcomes for the first time in China. We conducted this study in a representative sample from 15 provincial administrative regions in China. Therefore, we can generalize the results to female nurses of the whole country. Our findings emphasize the great importance of the policy for occupational hazard control among nurses in China, where there are tens of millions of female nurses.

There were also some limitations in our study. First, one obvious bias arose from the variety of occupational exposures and adverse pregnancy outcomes. Like other occupational epidemiology studies, information on occupational exposures and adverse pregnancy outcomes were self-reported in this study and based on questionnaire surveillance data rather than field monitoring data or work records. Nevertheless, we found significant associations between adverse pregnancy outcomes and expected risk factors. Additionally, the prevalence of most adverse pregnancy outcomes was higher among nurses than expected. Second, unlike the work during the normal period, pregnant females always took care of occupational exposures like prolonged standing or carrying heavily frequently. We had no data on whether nurses worked part-time or full-time during pregnancy. Hence, the risk of prolonged standing or carrying heavily frequently on pregnancy outcomes might be biased. Third, as it was a cross-sectional study, we measured both occupational exposures and adverse pregnancy outcomes at the same time. Therefore, we need to depend upon the responses given by the participants, and there may be a chance of recall bias. In other words, we could not establish the causality between them. However, although our findings of causal interpretation were not definite due to the nature of the cross-sectional study, there might be a biological rationale for our results. Previous cohort studies [11] revealed the associations between occupational exposures and adverse pregnancy outcomes. Further cohort study based on extensive population data is needed to investigate the causal relationship between occupational exposures and adverse pregnancy outcomes. Furthermore, we suggest that the Theory of Reasoned Action and the Health Belief Model should be used to explain the association between perception and action and then illustrate the reason for controlling and preventing occupational exposures among female nurses.

In summary, occupational exposures could account for the risk of adverse pregnancy outcomes among nurses in China. Regulations to reduce risk factors of adverse pregnancy outcomes and protect nurses from occupational exposures may be especially crucial for nurses of reproductive age. In detail, we strongly recommend policymakers develop guidelines on work-related counseling programs to mitigate occupational hazards for female nurses [52]. Hospital managers should take control measures and develop organizational policies to reduce exposure to occupational hazards and to improve pregnancy outcomes among female nurses. Nursing associations are also responsible for initiating workplace health policy development in hospitals.

### Supplementary Information

**Additional file 1:** Sensitivity analysis by excluding nurses from primary medical and health institutions.** Figure.** Adjusted regression models were displayed, controlling for age, marriage, and education level; LL: lower level of 95% confidence interval; UL: upper level of 95% confidence interval.

## Data Availability

The datasets generated during and/or analyzed during the current study are available from the corresponding author on reasonable request.

## Abbreviations

CIs: Confidence intervals
IQ: Interquartile rage
LL: Lower level of 95% confidence interval
UL: Upper level of 95% confidence interval
ORs: Odds ratios
