# Supplementary figures and images for: Associations between maternal occupational exposures and pregnancy outcomes among Chinese nurses: a nationwide study
Source: Reprod Health. 2023 Oct 31;20:161. doi: 10.1186/s12978-023-01704-x (PMC10617240; doi:10.1186/s12978-023-01704-x)

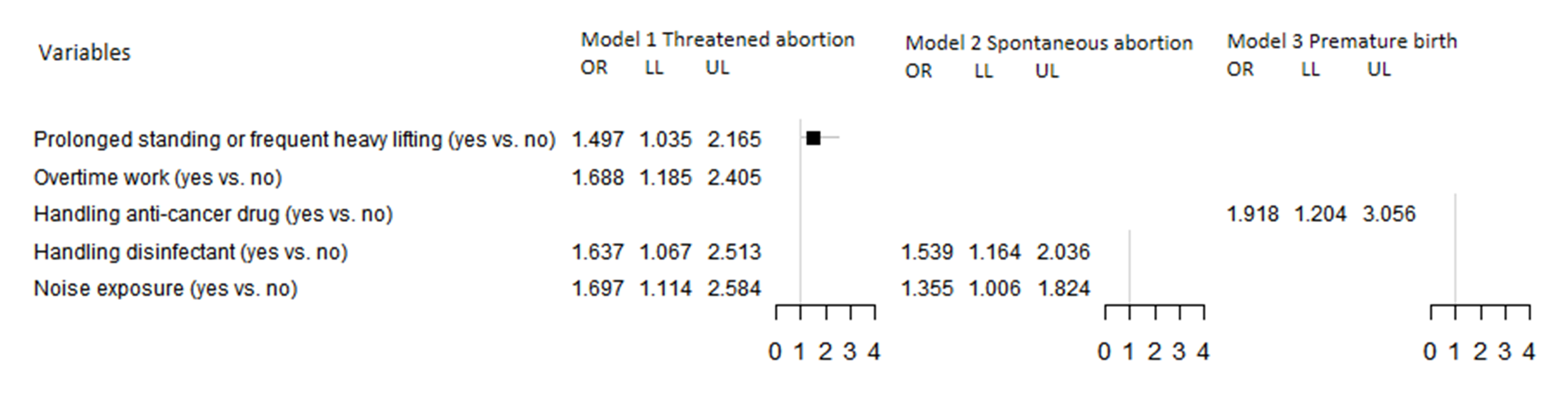

Supplement: Supplementary file 1 — Additional file 1: Sensitivity analysis by excluding nurses from primary medical and health institutions. Figure. Adjusted regression models were displayed, controlling for age, marriage, and education level; LL: lower level of 95% confidence interval; UL: upper level of 95% confidence interval. [file 12978_2023_1704_MOESM1_ESM.tif]
